# Emerging role of low-frequency somatic mutations in cancer relapse: from early detection to precision oncology

**DOI:** 10.3389/fonc.2026.1772616

**Published:** 2026-02-24

**Authors:** Eunsoo Kim, Gu Seob Roh, Seong Gyu Kwon

**Affiliations:** 1 College of Medicine, Gyeongsang National University, Jinju, Republic of Korea; 2 Department of Anatomy, College of Medicine, Metabolic Dysfunction liver disease Research Center, Institute of Medical Science, Gyeongsang National University, Jinju, Republic of Korea; 3 Department of Anatomy and Convergence Medical Science, College of Medicine, Institute of Medical Science, Gyeongsang National University, Jinju, Republic of Korea

**Keywords:** cancer relapse, low-VAF, next-generation sequencing, somatic mutation, ultra-deep sequencing

## Abstract

Somatic mutations with low variant allele frequencies offer a highly sensitive lens for detecting cancer relapse driven by diverse causes, including clonal evolution and therapy resistance. Advances in next-generation sequencing have enabled robust subclonal variant identification that typically fall below conventional detection limits, supporting a comprehensive understanding of individual molecular profiles that can lead to relapse. These low-level alterations frequently emerge before clinical or radiological relapse and can inform response-adaptive treatment decisions. This review integrates the current biological and technical insights into low-frequency mutations and evaluates their emerging roles in tumor relapse management and precision oncology.

## 1 Introduction

Tumor relapse poses a persistent challenge to cancer management and often contributes to poor long-term outcomes (1, 2). Despite advances in tumor treatment, methods for predicting and preventing relapse remain relatively unexplored. Cancer relapse is a complex genomic phenomenon driven by various factors, such as residual disease and malignant mutation-containing clones (3, 4).

Despite diverse treatment options for eliminating tumors, a small number of cancer cells remain unremoved and relapse is induced (5). Minimal residual disease (MRD) refers to these small clones, and is widely used in cancer management. Achieving MRD negativity is crucial for the overall cancer treatment course (6, 7).

Circulating tumor cells (CTCs) and circulating tumor DNA (ctDNA) are critical indicators of cancer relapse (8). CTCs are viable cancer cells released into the bloodstream and may contribute to the recurrence of cancer. In contrast, ctDNA comprises tumor-derived DNA fragments released from cancer cells and serves primarily as a biomarker reflecting tumor burden (9, 10).

Somatic mutations, defined as post-zygotic mutations in individual cells at any point in life, are among the most common causes of cancer (11, 12). Somatic mutations can exist in normal cells in a benign state, but can also accumulate with natural aging and develop into cancer (12–14).

The variant allele frequency (VAF) concept has been introduced as a tool for understanding somatic mutations in cancer using next-generation sequencing (NGS) (15, 16). The VAF is the proportion of sequencing reads supporting a given variant relative to the total number of reads covering the allele.

In oncology, a high VAF generally indicates a dominant clone or large tumor burden, whereas low VAF mutations reflect subclone populations, MRD, or early relapse signals (17). NGS enables highly sensitive and accurate measurement of VAF across a wide genomic range. VAF quantification using NGS provides critical insights into cancer diagnosis and treatment (15).

Furthermore, low VAF mutations, often defined as variants with VAFs of < 5% or 10%, have a unique position in cancer genomics. Historically, these alterations have often been regarded as sequencing artifacts or clinically irrelevant noise, primarily because of sensitivity-related limitations of the technology (18, 19).

However, developing precise bioinformatics pipelines has revolutionized mutation analysis and revealed that low VAF mutations can represent small cancer cell populations that are often undetected by traditional assays (20). This has transformed low-VAF mutations into critical components of cancer relapse management. Therefore, frameworks that distinguish between clinically relevant mutations and technical noise are required.

**Figure 1:**
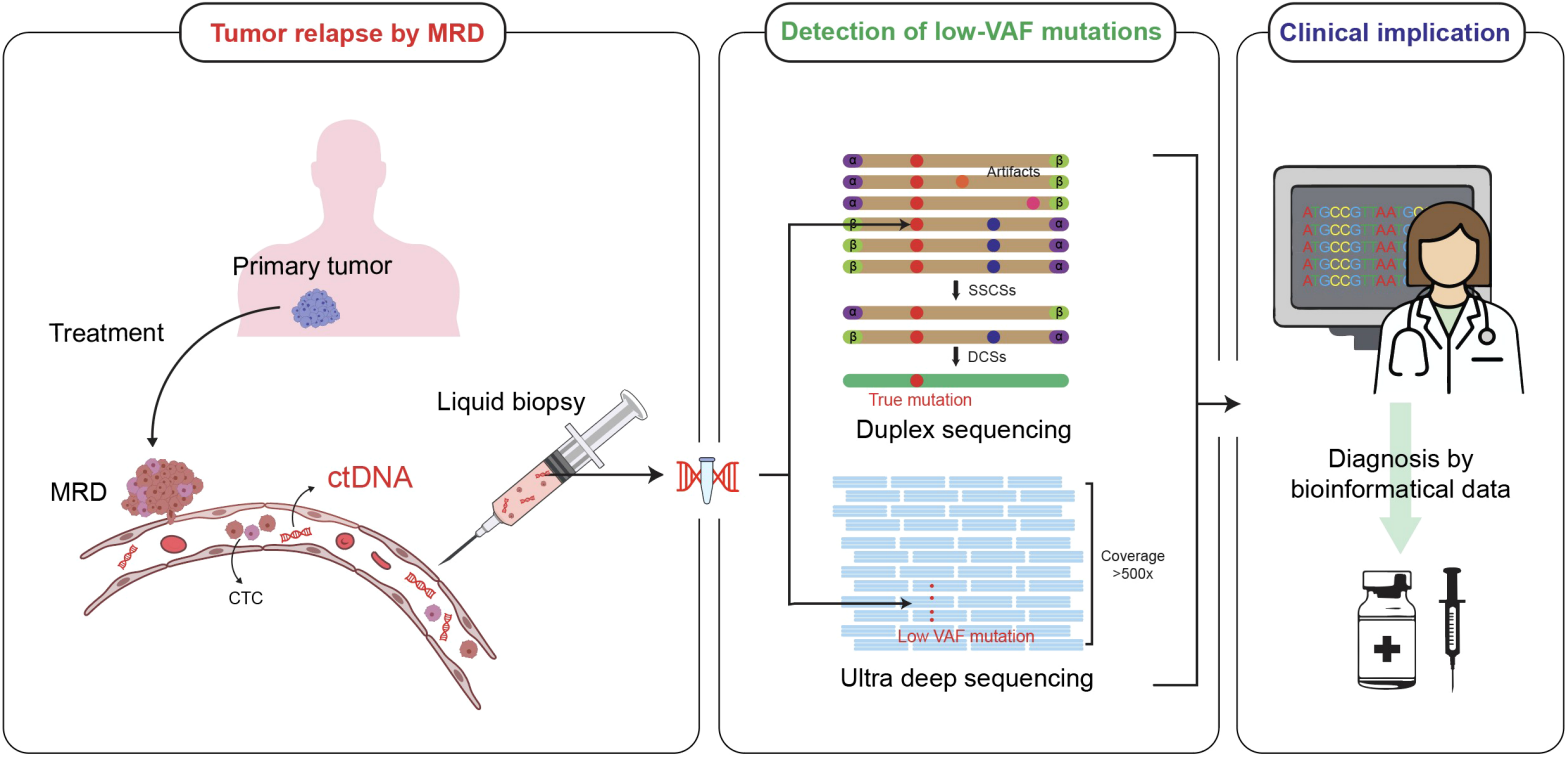
Clinical utility of detecting low-VAF mutations in post-treatment MRD via liquid biopsy. Posttreatment MRD is a potential source of metastasis mediated by CTCs and ctDNA. Liquid biopsy enables the isolation and analysis of these biomarkers to detect low VAF mutations using advanced sequencing technologies. This genomic profile provides insights into diagnosis and therapeutic decision-making.

This review aims to consolidate the current knowledge on the role of low-frequency mutations in cancer relapse diagnosis and treatment. By examining studies that associate VAF with cancer relapse, we explore the clinical significance of molecular monitoring systems and low-VAF mutations in managing tumor relapse (**Figure 1**).

## 2 Relapse statistics of 11 major cancer types

Cancer relapse is a considerable challenge in oncology. Despite enhanced treatment methods, including surgery, chemotherapy, radiotherapy, and immunotherapy, relapses remain unresolved. Patients experiencing recurrence face limited treatment options and adverse effects of therapies, resulting in a diminished quality of life and prognosis (21, 22).

### 2.1 Breast cancer

Breast cancer in women has become the second most diagnosed cancer worldwide, representing the fourth leading cause of cancer-related mortality in 2022. Breast cancer accounts for 11.6% of the total cancer incidence globally, with 2.3 million new cases and 665,684 deaths as of 2022 (23). A large meta-analysis reported that after stopping 5-year adjuvant endocrine therapy, ER-positive early breast cancer continued to recur during years 5–20, ranging from 10% to 41% across different TN groups (24). These late events would correspond with the expansion of small persistent subclones, emphasizing sensitive monitoring for low VAF detection.

### 2.2 Colorectal cancer

In the U.S., 107,320 cases of colon cancer and 46,950 cases of rectal cancer are estimated to occur by 2025 (25). Approximately 903,000 deaths from colorectal cancer occurred worldwide in 2022, ranking third in incidence and second in mortality (23). Colorectal cancer relapse threatens patient survival. A cohort of 2475 colon cancer patients demonstrated that right-sided colon cancer (RCC) (7.35%, CI 6.55–8.25) has a worse mortality rate than left-sided colon cancer (LCC) (5.32%, CI 4.57–6.20) (26). Furthermore, perforated colorectal cancer (PCC) is associated with inferior relapse outcomes compared with non-perforated colorectal cancer (27–29). High relapse risk is mainly attributed to leakage of tumor-related content into the peritoneum (27).

### 2.3 Pancreatic cancer

Pancreatic cancer has the highest mortality rate among all types of cancer. Regardless of tumor stage, the five-year survival rate in the United States is estimated to reach 13% by 2025 (25). An estimated 467,000 deaths occurred from 510,000 incidents worldwide in 2022 (23). The prognosis of pancreatic cancer is the poorest among all types of solid cancers, and selecting patients suitable for surgery is challenging (30, 31). Patients who can undergo curative surgery account for only 15%–20% of all patients with pancreatic cancer (32, 33). Relapse remains common even after surgery or aggressive therapy (32, 33).

### 2.4 Liver cancer

In 2020, liver cancer was the sixth most diagnosed cancer and the third leading cause of cancer-related mortality worldwide in 2020 (25). In 2022, 865,200 new cases and 757,900 deaths are estimated worldwide (23). A meta-analysis of 125 hepatocellular carcinoma studies demonstrated a pooled relapse rate of 17% and a mortality rate after recurrence of 9% (34). In addition, a cohort of hepatoblastoma patients revealed that combined relapse (simultaneous local and distant recurrence) was associated with particularly unfavorable outcomes compared to sole metastatic relapse (35).

### 2.5 Non-small cell lung cancer

In a population study in the U.S., non-small cell lung cancer (NSCLC) accounted for approximately 84% of all lung cancer subtypes between 2010 and 2017, and its prognosis was poor (36). In a study of 775 patients with NSCLC who underwent curative surgery, 133 experienced relapse. The two-year OS of patients who relapsed was 37%, and 83% of patients who underwent limited surgery relapsed within a year (37).

### 2.6 Prostate cancer

Prostate cancer is one of the most frequently diagnosed cancers among men, with over 1,446,680 cases and 396,700 deaths worldwide by 2022 (23). In the United States, prostate cancer-related mortality has steadily declined over the past decades (25). However, prostate cancer relapse is a common phenomenon. According to two population studies, 30% of patients who underwent radical prostatectomy relapsed within 10 years (38, 39).

### 2.7 Blood cancer

The global incidence and mortality of blood cancer have steadily increased over the last 30 years (40). In 2022, approximately 553,000 cases of non-Hodgkin’s lymphoma, 486,700 cases of leukemia, and 187,700 cases of multiple myeloma occurred globally (23). The incidence rates are predicted to increase annually by 1.7% for multiple myeloma, 0.79% for leukemia, and 1.65% for non-Hodgkin’s lymphoma from 2020 to 2030 (41). Relapse occurs frequently after transplantation, leading to a poor prognosis. In a cohort of 1080 patients, 351 relapsed during a four-year follow-up period, with a 19% three-year OS rate after relapse (42).

### 2.8 Stomach cancer

Stomach cancer accounts for 968,350 new cases in 2022 globally, with the fifth highest total cancer incidence and mortality (23). Two studies demonstrated that 42% and 46.5% of patients with stomach cancer who underwent curative surgery relapsed (43, 44). Postoperative relapse occurring within two years of surgery seriously affects patient survival (44).

### 2.9 Kidney cancer

The global incidence of kidney cancer is expected to increase from 160,000 cases in 1990 to 390,000 in 2021 (45). Although the overall mortality rate is relatively lower than that of other cancer types, renal pelvic tumors can be fatal (25, 46). In a study of 143 kidney cancer patients who underwent renal transplantation, 13 patients relapsed, and only 3 of them survived at the end of the follow-up (47). Additionally, a study on renal cell carcinoma demonstrated that 30% of patients experienced relapse after curative treatment (48).

### 2.10 Uterine cancer

Uterine cancer recurrence results in inferior outcomes compared to non-recurrent cases, especially when the time to relapse after surgery is short. A study of 35 endometrial cancer relapse patients demonstrated that the three-year survival rate was 64.9% in patients with one relapse site and 39.2% in those with multiple relapse sites (49, 50). Multi-site relapse can arise through multiple subclones or monoclonal seeding followed by diversification. Additionally, a study of 1503 patients with endometrial cancer presented the histological type, grade, and secondary radical surgery as meaningful factors for post-relapse survival (51).

### 2.11 Brain cancer

According to a 30-year epidemiological study, the global burden of central nervous system cancer has steadily increased from 2,831,075 new cases in 1992 to 3,420,786 in 2021. The number of deaths from brain cancer increased by 80.62% (52). Relapse of cancer related to the brain can be damaging. Central nervous system involvement in mature T- and NK-neoplasms results in a significantly high mortality rates (53). Similarly, glioblastoma, the most frequent form of brain cancer, has a poor prognosis, with a 15-month median OS upon relapse (54).

Relapse rates vary between cancer types, primarily because of differences in tumor biology or specific mutations. Different cancers and subtypes can have distinct genetic characteristics (55, 56). Several tumor subtypes tend to have high mutation burdens, malignant mutations, and relapses (57).

## 3 Advances of NGS and oncology application

### 3.1 NGS overview

NGS development represents a pivotal step in the history of genomics (58). Early sequencing methods, such as Sanger sequencing, provided the foundation for interpreting DNA but had limitations, such as low throughput and time-consuming protocols (59). With completion of the Human Genome Project, the need for fast and scalable sequencing has become evident (60, 61). This demand has driven the emergence of NGS, which has enabled parallel sequencing of massive amounts of data and improved the efficiency of genome analysis (62).

NGS sequences millions of short DNA fragments in parallel, followed by computational alignment to reconstruct the genome (63, 64). This fundamental shift to parallel processing has helped address broader purposes such as whole genome sequencing (WGS), whole exome sequencing (WES), and targeted sequencing (65, 66). These widespread applications have benefitted clinical medicine, where NGS is increasingly used to diagnose diseases, guide treatment, and monitor disease progression (67, 68).

### 3.2 NGS methodology in cancer diagnosis and surveillance

Enhanced NGS technologies are advantageous in oncology, where tumor heterogeneity and mosaicism pose challenges for diagnosis and treatment (62, 69). By capturing mutations across a wide range, NGS has reinforced our understanding of the genetic profiles of patients (67, 70).

For relapse monitoring, NGS assays often adopt either tumor-informed tracking of patient-specific variants or panel-based tests using fixed panels targeting known recurrent alterations (66). While fixed panels often prioritize previously identified mutations, tumor-informed monitoring can find patient-specific variants found at diagnosis, including non-hotspot and private variants, enabling individualized surveillance.

Accumulating evidence indicates that NGS is superior to other molecular assays and nonmolecular methodologies for diagnosis (71). NGS detects cancer mutations at lower frequencies that may be missed using traditional methods, including pathological tests (66, 72). Likewise, a study of 1266 lung cancer cases compared the accuracy of NGS and clinicopathological methods and demonstrated that the latter had lower accuracy than NGS, with one-third of the errors. However, NGS can accurately predict prognosis, contributing to an improved OS (72).

Furthermore, several studies comparing the accuracy of NGS and PCR have presented NGS as a better method for detecting cancer mutations (73, 74). In a study focusing on hotspot mutations in tumors, NGS-based tests detected eight mutations that were not detected using PCR (73). Another study analyzed the ability of NGS to detect EGFR, KRAS, and BRAF mutations and showed that NGS detected seven mutations that PCR could not detect (74).

### 3.3 Ultra-deep sequencing to detect low-frequency mutations

As NGS platforms and methodologies have advanced, the identification of mutations with low VAFs has become reliable (75, 76). For example, the introduction of blocker displacement amplification, which uses qPCR and Sanger sequencing to filter and confirm mutations, achieved a 0.1% limit of detection (LoD) (77). Duplex sequencing labels both strands of a DNA molecule with distinct molecular indices, sequences them independently, and accepts a variant if an identical change is observed in both complementary strands, to suppress artifactual errors (78).

Error-corrected NGS uses consensus sequences selected by multiple rounds of sequencing to minimize errors and unique molecular identifiers to label mutations (79). Hotspot cancer mutations can also be detected using targeted ultra-deep sequencing. A study focusing on hotspot mutations achieved 97.1% sensitivity and 97.9% specificity for detecting BRAF with a LoD of 0.025 (80). Accelerated developments in molecular biology have paved the way for precision medicine in clinical settings, including monitoring and personalized treatment.

## 4 Clinical implications of low-frequency mutations in cancer relapse

### 4.1 Biological sources of low-frequency mutation

Technical progress has uncovered the burden of low-VAF mutations in tumors, highlighting their potential for cancer relapse prevention and treatment (81, 82). A previous study sequenced 300,000 tumor samples and revealed that 29% of patients retained more than one mutation in 10% of cases, and 16% had mutations in less than 5% of cases (83). Detectability of low-VAF variants is primarily dependent on sample quality and assay sensitivity, with varying clinical severity based on patient context.

Low-VAF mutations can be detected in diverse sources (84, 85). ctDNA is widely used for solid cancers because blood is easily accessible and less invasive than tissue biopsies (86–88). CtDNA can be detected across a broad VAF spectrum that depends on disease burden, tumor fraction, and assay sensitivity, ranging from ultra-low levels to higher, dominant status (89). Therefore, sampling frequency and duration are not standardized across cancer types, highlighting the necessity for tailored strategies.

In addition, CTCs are being increasingly explored as key biomarkers of cancer relapse (90). Single-cell DNA analysis can be used to detect mutations in CTCs and to predict cancer relapse (91). A previous study analyzed CTCs from metastatic breast cancer patients and detected PIK3CA mutations at a frequency of 1% (92).

Mutations in blood cancers can be detected in the bone marrow, peripheral blood, or lymphocyte tissues (93, 94). One study demonstrated that targeted NGS of cell-free DNA (cfDNA) detected several mutations that were not found solely in liquid tissue, usually extracted from the bone marrow (95).

Furthermore, extracellular vesicle DNA (EV-DNA) can be used for mutation profiling (96). An extracellular vesicle (EV) is a particle enclosed in a membrane containing DNA, RNA, and proteins. As EVs play a significant role in the pathological mechanisms of cancer and are stable due to their larger size, they have the potential to be used as a cancer biomarker (96–98). A study comparing the frequencies of tissue DNA and EV-DNA demonstrated that mutations detected by EV-DNA (<5%) had lower VAFs than those detected by tissue DNA (10%–25%). However, the relationship between tissue DNA and EV-DNA can depend on diverse elements, requiring cautious interpretation (99). At present, there is no consensus on which biomarker should be prioritized over others. EV-DNA may provide complementary information in specific cases, but it is not yet established as a universal method for cancer screening.

### 4.2 Application of low-VAF mutations in the diagnosis of cancer relapse

#### 4.2.1 Early diagnosis and prevention of cancer relapse

NGS diagnoses cancer relapse earlier than traditional imaging techniques such as computed tomography (CT) or magnetic resonance imaging (MRI). Advanced molecular approaches, including duplex sequencing and error-corrected NGS, can be used to detect mutations in ctDNA, CTCs, and EV DNA.

In a study that developed a method applying targeted sequencing to phased variants of ctDNA, the lowest VAF was 0.000094% in patients with stage I NSCLC. These mutations are highly predictive of cancer relapse, enabling diagnosis 5–10 months earlier than radiology (100). Another study examining the relationship between MRD and acute myeloid leukemia relapse using error-corrected sequencing demonstrated that MRD positivity predicted recurrence with high sensitivity. Targeted ctDNA sequencing of 29 genes was performed using bone marrow or peripheral blood samples. MRD was detected in 35% of patients using duplex sequencing, and the relapse risk was 8.8 times higher than that in patients who are MRD-negative (101).

Furthermore, longitudinal monitoring can help diagnose early relapses. A previous study analyzed the ctDNA of 130 patients with colorectal cancer throughout treatment and surveillance. Deep sequencing with > 10,000 coverage has been applied, supporting relapse prediction up to 16.5 months earlier than traditional methods (102).

CTCs and EV-DNA play crucial roles in recurrence surveillance and diagnosis (103, 104). One study that applied single-cell sequencing to bulk CTC samples from patients with small-cell lung cancer demonstrated that low-VAF mutations in CTCs corresponded with mutations in the original tumor. For instance, the VAF of a CLCA2 mutation is approximately 4% in bulk CTCs and 7% in primary tumor tissue (105). Another study conducted EV-DNA analysis in early-stage NSCLC patients and captured EGFR mutations in 38 EV-DNA samples, with VAFs ranging from 0.1% to 1.3%, suggesting that EV-DNA is an indicator of cancer relapse (106).

#### 4.2.2 Detection of *de novo* cancer mutations during recurrence

Cancer biomarkers can detect *de novo* mutations with the potential for relapse, which often occur during therapy or monitoring. Discovering *de novo* mutations requires high sensitivity as they typically emerge at low frequencies.

A study analyzing cfDNA from NSCLC patients using targeted sequencing revealed *de novo* oncogenic mutations with high sensitivity and specificity. Driver and resistance mutations were detected in several patients. Most variants, including hotspot mutations such as EGFR and KRAS, were detected at VAFs below 10% (107).

A case report of a 66-year-old breast cancer patient with metastasis to the bone and liver involved *de novo* mutation analysis of the primary tumor site, the liver metastatic site, and four plasma samples. A fraction of mutations with VAFs below 5% underwent clonal evolution under selective pressure, expanding to dominant fractions ranging from 26-68%. Accordingly, some low-VAF mutations can later be found as dominant clones (108).

Additionally, serial DNA monitoring of CTCs in patients with CRC detected new missense mutations. A patient who received irinotecan and cetuximab treatment developed a *de novo* SMRCB1 mutation with a CTC frequency of 7.22%. Similarly, another patient developed a PIK3CA mutation at a 7.05% frequency (109).

#### 4.2.3 Cancer relapse risk stratification with high sensitivity

Stratification of cancer relapse risk can help identify high-risk patients who can benefit from intensive monitoring and treatment. Similarly, low-risk patients, who do not require as much screening as high-risk patients, can avoid overtreatment. The accurate measurement of cancer variants can guide tailored treatment adjustments.

A study involving 137 patients with large B-cell lymphoma conducted risk stratification of PET-negative patients using ctDNA. Although classified as a low-risk group based on PET scans, the ctDNA-positive group exhibits a 30-fold increased relapse risk and worse progression-free survival (PFS) (110).

A study conducted WES to monitor MRD in seven high-risk epithelial ovarian cancer patients, five of whom were MRD-positive at baseline, with a median VAF of 2.79%. Two patients were MRD positive after surgery, with VAFs of 0.04% and 0.09%, respectively. Two patients were reclassified in the high-risk group and received additional chemotherapy to prevent relapse (111).

By comparing the effectiveness of risk stratification using clinicopathological markers and ctDNA, risk assessment using clinicopathological methods tended to be less sensitive. However, ctDNA enables sensitive measurements, thus reducing overtreatment by adaptive therapies based on individual genetic profiles (112).

Furthermore, EV-DNA demonstrated higher sensitivity than ctDNA in predicting colorectal cancer recurrence. Measurement of KRAS mutations using EV-DNA showed better prediction than ctDNA and tissue biopsy. Moreover, applying NGS to EV DNA is more effective than simply measuring DNA concentration (113).

### 4.3 Application of low-VAF mutations in relapsed cancer treatment

Low-VAF mutations can indicate therapeutic resistance and assist in early detection before progression. Precise monitoring of subtle mutational changes can provide a basis for personalized treatment. When low-VAF mutations persist or increase, they may signal residual disease, guiding treatment adjustments such as drug switching or extended treatment duration (114). Moreover, low-VAF mutations after primary treatment can inform decisions regarding adjuvant therapy and long-term surveillance.

In a study of NSCLC patients treated with an immune checkpoint blockade, serial ctDNA analysis supported the adjustment of treatment intensity. The median VAF of cancer mutations was 1.87%, ranging from 0.09%–34.7%. Patients without molecular clearance had a substantially shorter PFS than those in the ctDNA-negative group (115).

Sequencing plasma samples from patients with NSCLC receiving PD-1 inhibitors revealed that subtle changes in ctDNA signaled the emergence of immune-escaping clones. Increases in low-frequency variants related to immune escape precede disease progression by months, emphasizing the importance of regular screening (116).

In urothelial bladder cancer, ctDNA profiling enables real-time treatment adjustments by reflecting therapeutic responses. In this cohort, decreased ctDNA levels during chemotherapy predicted pathological downstaging. Among ctDNA-positive patients before or during therapy, ctDNA clearance was associated with complete remission, whereas persistent ctDNA levels implied a higher risk of recurrence (117).

Moreover, a study addressing the efficacy of drug switching monitored patients with advanced breast cancer receiving first-line aromatase inhibitors and palbociclib. Patients with emergent ESR1 mutations were randomized to either continue the current therapy or switch to fulvestrant. The molecular-guided drug switch was shown to improve PFS (median 11.9 vs 5.7 months) (118).

A study that used EV-DNA to detect therapy resistance in patients with NSCLC after first-line EGFR-TKI treatment demonstrated that most patients acquired resistance within 1–2 years, and T790M was the key mutation guiding the switch to osimertinib. Combining cfDNA with EV-DNA improves the sensitivity of detecting therapy-resistant variants (119).

A study on early stage breast cancer demonstrated that deep ctDNA sequencing can guide adjuvant therapy decisions. Using patient-specific multiplex sequencing, baseline ctDNA was detected in 32 patients, with a median VAF of 0.11%. After therapy, patients diagnosed with pathological complete remission showed near clearance, with VAFs < 0.003% (120).

The development of sensitive sequencing to detect low-VAF mutations has enabled precise clinical decision-making across various stages of cancer. Quantifying low-VAF alterations allows for earlier identification of recurrence compared to radiological or pathological methods. This finding also supports proactive interventions and treatments. NGS can guide response-adaptive decisions during cancer treatment and maintenance phases. The integration of sensitive mutation tracking into routine relapse management will lead to a personalized and sensitive approach to the detection and treatment of cancer.

## 5 Conclusion

Enhanced capability to detect low-VAF mutations represents a milestone in oncology, moving toward precise and personalized cancer care. Cancer is intrinsically prone to relapse due to its mutational diversity. The advent of ultra-deep sequencing has enabled the identification of low-VAF mutations that were previously missed, opening a new window to understand cancer biology and improving patient prognosis.

The clinical applications of low-VAF mutations span the entire process of cancer relapse management. Sensitive sequencing provides a basis for therapeutic intervention when tumor burden is minimal, often predicting relapse months before imaging and radiology can. In treatment, low-VAF mutations can inform treatment directions, including drug switches and therapy intensity adjustments. Importantly, clinical actions triggered by low-VAF mutation profiling are context-dependent. Some alterations would primarily support risk stratification and intensified surveillance, whereas others may suggest treatment adaptation.

However, several challenges should be addressed before low-VAF mutations are fully utilized in routine practice. Tumor heterogeneity from continuous mutagenesis and clonal evolution hinders the detection of low-frequency mutations (121). This spatial and temporal variation implies that mutations from one site or time point may not reflect the complete disease landscape (122).

Additionally, standardized laboratory protocols are insufficient, causing inconsistent results across laboratories using distinct bioinformatic pipelines. Establishing guidelines would improve inter-laboratory compatibility (123). Standardization in clinical decision-making can also help define when to prioritize one biomarker over another based on specific patient conditions, specimen types, and mutation assessment methods.

The value of low-VAF mutation lies in its potential to transform cancer relapse management into truly personalized care by guiding precise decision-making. These advances will improve cancer survival and prognosis by tailoring strategies based on molecular profiles.
